# Augmenting *Nesidiocoris tenuis* (Nesidiocoris) with a Factitious Diet of *Artemia* Cysts to Control *Bemisia tabaci* (Gennadius) on Tomato Plants under Greenhouse Conditions

**DOI:** 10.3390/insects12030265

**Published:** 2021-03-21

**Authors:** Takeshi Saito, Motonori Takagi, Toshiyuki Tezuka, Takashi Ogawara, David Wari

**Affiliations:** 1 Horticultural Research Institute, Ibaraki Agricultural Center, 3165-1 Ago, Kasama City, Ibaraki 312-0292, Japan; tohop1341398@gmail.com (T.S.); mo.takagi@pref.ibaraki.lg.jp (M.T.); 2 Ibaraki Agricultural Academy, 4070-186 Nagaoka, Ibaraki Town, Ibaraki 311-3116, Japan; 3 Research and Development Division, Agri-Soken Inc., 2629-1 Numata, Inashiki City, Ibaraki 300-0506, Japan; t_tezuka@agri-insect.com

**Keywords:** *Artemia* cysts, *Bemisia tabaci*, factitious diet, high fructose corn syrup, honey, Integrated Pest Management (IPM), natural predator, *Nesidiocoris tenuis*

## Abstract

**Simple Summary:**

*Nesidiocoris tenuis* (Hemiptera: Miridae) is an important biocontrol agent of several key arthropod pests, such as greenhouse and tobacco whiteflies, the South American tomato pinworm, thrips, plant mites, and other pests in the greenhouse. However, optimizing *N. tenuis* utilization in the greenhouse to control greenhouse pests such as whiteflies still needs further studies, especially in Japan. Here, we showed that factitious supplementary dietary in the form of *Artemia* cysts enhanced with high fructose corn syrup and honey, and delivered using a hemp rope could promote *N. tenuis* proliferation and spread among tomato plants. *Nesidiocoris tenuis* spread among tomato plants therefore, can maintain whitefly (*Bemisia tabaci*) eggs and nymph numbers at minimum in the greenhouse conditions.

**Abstract:**

Natural predators such as *Nesidiocoris tenuis* are known for their role in managing greenhouse pests. However, techniques in maximizing the biological control potential of *N. tenuis* under field conditions are still lacking. We evaluated under greenhouse conditions the prospects of *Artemia* cysts enhanced with high fructose corn syrup and honey, and delivered using hemp strings (hemp rope) as supplementary factitious dietary in augmenting the proliferation and spread of *N. tenuis* on tomato plants. Results showed that *N. tenuis* supplemented with hemp rope could establish, proliferate and disperse among tomato plants compared to the *N. tenuis* supplemented with banker plants. Even though *N. tenuis* proliferated exponentially on banker plants, their movement and relocation to tomato plants, as expected, were only congested on tomato plants near the banker plants. However, as the survey continued, they relocated to the rest of the tomato plants. Furthermore, the number of *Bemisia tabaci* eggs and nymphs, a serious greenhouse pest of tomato, was observed to be significantly reduced in hemp rope greenhouse compared to banker plants and the negative control (no pest control system) greenhouses. This study, therefore, establishes foundational data on the usage of *Artemia* cysts enhanced with isomerized sugar (high fructose corn syrup) and honey under greenhouse conditions as factitious supplementary dietary in supporting *N. tenuis* establishment and spread, traits that are essential towards development of whitefly Integrated Pest Management (IPM) system. enhanced with isomerized sugar (high fructose corn syrup) and honey.

## 1. Introduction

Tomato (*Lycopersicon esculentum* Mill.) production, almost always under the greenhouse conditions across the whole of Japan was estimated at around 724,200 tons in 2018 with 11,800 hectares of total land use, yielding productivity of around 6140 kg per 0.1 ha [1]. Tomato production, however, is always constantly been threatened by greenhouse pests. These major greenhouse pests include whiteflies (*Bemisia tabaci* Gennadius, 1889), thrips (*Thrips palmi* Karny, 1925), and tomato leafminers (*Tuta absoluta* Meyrick, 1917). Amongst these three major greenhouse pests, *B. tabaci* has been of great economic importance, not only in Japan but globally. *Bemisia tabaci* not only threatens through its herbivory damages but also vectoring viral diseases such as Tomato yellow leaf curl virus (TYLCV) and Tomato Chlorosis virus (ToCV), and the excretion of honeydew from the gut that encourages the growth of fungi. Per se, *B*. *tabaci* has been a major concern for most of the tomato growers in Ibaraki prefecture, where this study was conducted. Currently, in Ibaraki prefecture, *B. tabaci* management mostly relies on the application of insecticides. However, excessive use of insecticides may cause *B. tabaci* to develop resistance. To minimize excessive use of insecticides, biological control using naturally occurring enemies have been proposed.

The use of zoophytophagous predators as natural enemies to manage greenhouse pests has been gaining momentum and success around Europe and the Mediterranean basin [2]. In Japan, several studies have been reported on the use of *Nesidiocoris tenuis* (Reuter, 1895), a classical example of zoophytophagous predator, to control greenhouse pests such as whiteflies and aphids [3,4,5,6,7]. However, *N. tenuis* know-how in the management of greenhouse pests has been much of a deliberation. The essential practices such as release amount and the rate at which it may not cause any damage on tomato plants [8]; supplementation with banker plants [9]; spectral light wavelengths as a means of natural enemy attraction mechanism [5] and the use of *Ephestia kuehniella* Zeller (Lepidoptera: Pyralidae) eggs and Brine shrimp (*Artemia* spp., Anostraca: Artemiidae) cysts as alternative factitious dietary [7] are few that have been suggested to augment *N. tenuis* as biocontrol agent. The latter has been determined in vitro [7]; however, field studies have been lacking.

Brine shrimp eggs are potentially useful as a low-cost alternative and supplementary dietary for biological control agents [7]. Various in vitro studies have reported the use of brine shrimp eggs as alternative and supplementary dietary for natural enemies such as *Adalia bipunctata* (Linnaeus, 1758) [10], *Amblyseius swirskii* (Athias-Henriot, 1962) [11], *Macrolophus caliginosus* (Wagner, 1951) [12], *M. pygmaeus* (Rambur, 1839) [13], *Harmonia axyridis* (Pallas, 1773) [14,15], *Orius laevigatus* (Say, 1832) [16], and *O. strigicollis* (Poppius, 1915) [17]. With extensive studies elucidating the role of brine shrimp eggs in promoting growth and development of natural enemies in vitro, particularly with their fecundity and survival, brine shrimps could be of significance in greenhouse or open field settings [15]. Therefore, in this study, we determine the role of *Artemia* cysts augmented with high fructose corn syrup and honey and delivered using a hemp rope along the rows of tomato plants in the greenhouse to promote *N. tenuis* propagation, proliferation, and spread among the tomato plants. Furthermore, we determine if the *N. tenuis* propagation, proliferation, and spread among the tomato plants can have an impact on controlling *B. tabaci* populations on tomato plants under greenhouse conditions.

## 2. Materials and Methods

### 2.1. Factitious Dietary, Banker Plants and Insect Rearing

*Artemia* cysts (*Artemia salina* (Linnaeus, 1758)) and *N. tenuis* were provided by Agri-Soken Inc. (Ibaraki, Japan). *Artemia* cysts were adhered to a 10 m long three-strand 3 mm thickness hemp string with a mixed solution of high fructose corn syrup and honey. The hemp string containing the *Artemia* cysts with high fructose corn syrup and honey is now referred to and hereafter used as the hemp rope and used as a factitious supplementary dietary for *N. tenuis* in the greenhouse tests. The hemp rope was kindly supplied by Agri-soken to facilitate this study. The ratio of *Artemia* cysts, high fructose corn syrup, and honey remains the sole property and trade secret of Agri-soken Inc.

Verbena cuttings (*Verbena terena* cv. Tapian) (Agri-soken Inc., Ibaraki, Japan), Sesame seeds (*Sesamum indicum*) (Sakata Seed Garden Center Ltd., Kanagawa, Japan) and Cleome plantlets (*Cleome spinosa*) (Proven Winners North America LLC., Sycamore, IL, USA) were propagated in the glasshouse at Ibaraki Horticultural Research Institute. *Nesidiocoris tenuis* is known to prefer these plants and can proliferate on these plants in high numbers in a short period [9].

*Nesidiocoris tenuis* populations were reared on verbena, sesame, and cleome at Ibaraki Horticultural Research Institute insectary rooms under optimized conditions (25 ± 1 °C, 65 ± 10% RH, and 16:8 L:D photoperiod). *Bemisia tabaci* populations were originally collected from capsicum fields in Kamisu City (Ibaraki Prefecture, Japan) in 2011 and were sparingly maintained on eggplants (*Solanum melongena*), kidney bean (*Phaseolus vulgaris* L.), and green bell pepper (*Capsicum annuum*) as a food source at Ibaraki Horticultural Research Institute insectary following the methods described by Wari et al. [18].

### 2.2. Greenhouse Preparations and Experiment Settings

**Figure A1:**
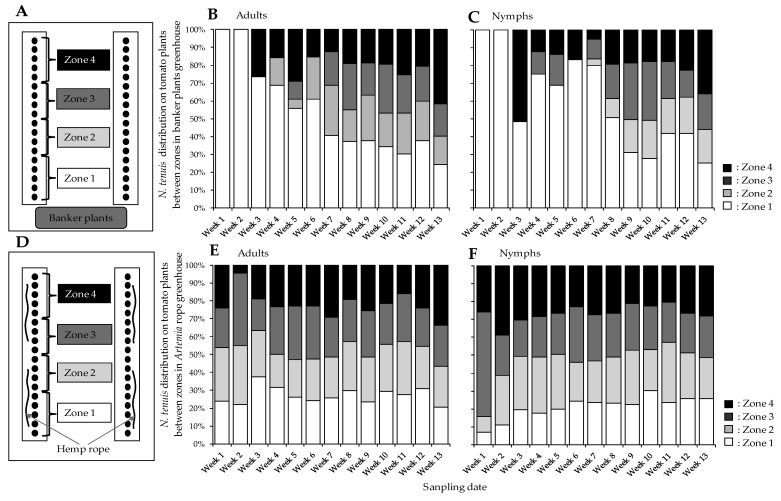
Greenhouse layout for banker plant treatment greenhouse (**A**) and hemp rope treatment greenhouse (**D**). Two rows of beds were prepared with 8 m each in a 10 m by 5 m vinyl house. Tomato plants were planted with a 40 cm spacing and divided into four zones, zones 1, 2, 3, and 4. For banker plant treatment, banker plants were planted on one end, adjacent to zone 1 (**A**). In the case of hemp rope treatment, four 2.5 m hemp rope containing *Artemia cysts* enhanced with high fructose corn syrup and honey were set on tomato seedling for each bed adjacent to each other (**D**). *Nesidiocoris tenuis* adults (**B**,**E**) and nymphs (**C**,**F**) distribution on tomato plants as a result of moving from banker plants or proliferating from hemp rope was surveyed. White bars represent the proportion of *N. tenuis* adults and nymphs in zone 1, grey bars for zone 2, dark grey for zone 3, and black bars for zone 4.

Field studies were performed in two consecutive years, 2019 and 2020. The experiments were conducted in the Horticultural Research Institute inside a 10 m by 5 m vinyl pipe greenhouse (See Appendix A Figure A1). Tomato Momotaro Peace cultivar, a TYLCV tolerant cultivar (Takii Seed Co., Ltd., Kyoto, Japan) was used in this study. Basic greenhouse preparations such as soil fumigation, plowing, rotary, fertilizer applications, bedding, and mulching were performed according to the tomato farming schedules practiced by Ibaraki Prefecture tomato farmers. In the case of this study, these basic greenhouse preparations were performed between April and May. Tomato seeds were sowed in early May, then two weeks later, transplanted onto vinyl pots (105 mm). After a month in vinyl pots, the 1.5 months old tomato seedlings were transplanted into the greenhouses. A total of 38 to 40 tomato plants per greenhouse were planted in two rows of beds (See Appendix A Figure A1). In the same week, banker plants (verbena, sesame, and cleome) used in propagating *N. tenuis*, were also planted in the greenhouse (negative control treatment, see below). *Bemisia tabaci* adults propagated in the Horticultural Research Institute insectary were released on tomato seedlings (two months after transplanting) at a rate of one *B. tabaci* adult per tomato plant. Two weeks after release of *B. tabaci*, *N. tenuis* was released onto tomato seedlings containing the hemp rope (for hemp rope treatment) and banker plants (for banker plants treatment) at the same rate, i.e., one *N. tenuis* (irrespective of the growth stage) per two tomato seedlings as recommended in the manual of NARO (National Agriculture Research Organization) manual [9]. Hemp rope was set on the same day *N. tenuis* was released. The assessment was started one month after the release of *N. tenuis*.

### 2.3. Experimental Treatment Settings

To examine if *Artemia* cysts enhanced with high fructose corn syrup and honey and delivered using a hemp rope could promote *N. tenuis* propagation, proliferation, and spread among the tomato plants in the greenhouse, thereby maximizing its predation potential, four test treatments were considered. These include (i) Hemp rope, (ii) Banker plants, (iii) Positive control, and (iv) Negative control treatment. In the Hemp rope treatment greenhouse, *N. tenuis* nymphs and adults were randomly released on tomato plants containing the hemp rope with rates as mentioned earlier. In a 10 m by 5 m vinyl pipe greenhouses, two rows of 8 m beds were raised as discussed above and hemp rope (2.5 m each) were set on each bed adjacent to each other to cover every tomato seedling (Figure A1A,D). Banker plants are widely reported to provide shelter, food sources, and medium for breeding and, therefore, are advantageous for *N. tenuis* propagation and proliferation. Thus, a banker plants treatment greenhouse was included to compare the movement and relocation of *N. tenuis*. In the banker plant treatment greenhouse, *N. tenuis* was released together with the banker plants (verbena sesame and cleome). Banker plants were planted at one end of the vinyl pipe greenhouse as per the recommendation by NARO [9] in integrating *N. tenuis* and banker plants to control *B. tabaci* under greenhouse conditions.

To evaluate the role of *N. tenuis* in managing *B. tabaci* in the greenhouse, we included both a positive and a negative control treatment. Positive control treatment greenhouse involved the use of synthetic insecticides to control *B. tabaci* while negative control had no means of any pest control measures. *Nesidiocoris tenuis* was not released in positive and negative control treatments. *Bemisia tabaci* was added in all treatments (i.e., hemp rope, banker plants, positive control, and negative control) to substantiate the role of *N. tenuis* when augmented with a factitious diet. As mentioned previously, this study was performed annually in two different years (i.e., 2019 and 2020) from April to October. Each annual year study was considered as replicates.

### 2.4. Sampling and Quantification of Test Subjects

In order to manage greenhouse pests that are almost always distributed among all the plants in the greenhouse, natural enemies must be scattered among all crops to maximize control impact. To determine if *N. tenuis* (both nymphs and adults) are scattered among the tomato plants in the greenhouse, we quantified the distribution of the *N. tenuis* individuals on all the tomato plants planted in the greenhouse. Tomato plants were grouped into clusters making four zones along the greenhouse. *Nesidiocoris tenuis* individuals on every tomato plants in each zone were quantified. *Nesidiocoris tenuis* individuals on tomato plants were quantified by visually inspecting each tomato plant in the greenhouse. *Nesidiocoris tenuis* nymphs and adults on the underside and topside of tomato leaves, as well as on the flower stalks and shoots, were also counted and recorded. To minimize errors in quantifying *N. tenuis* on tomato plants, as adults are prone to disperse when disturbed, disturbance of tomato plants were minimized. Furthermore, the entire counting was performed by one person for consistency and to keep track of the *N. tenuis* movement. *Nesidiocoris tenuis* nymph and adult counts for each zones (10~12 tomato plants per zone) were pooled together and the average determined to represent each zone. To determine the population dynamics of *N. tenuis* on tomato plants in the greenhouse throughout the study period, *N. tenuis* (both nymph and adult) counts from all tomato plants (40~48 tomato plants per greenhouse) were pooled together and the average calculated to represent the number of *N. tenuis* per tomato plant. The averages for each sampled week (among zones and all plants in the greenhouse) were treated as replicate for each treatment per annual study. *Nesidiocoris tenuis* on the banker plants was quantified by placing a 40 cm by 20 cm white bath container 10–15 cm beneath the banker plants and tapping ten times on the banker plants. *Nesidiocoris. tenuis* nymphs and adults that fell onto the white bath container were quantified and recorded. Sampling of *N. tenuis* on banker plants were performed separately for each banker plants, i.e., verbena, cleome and sesame.

*Bemisia tabaci* eggs, nymphs, and adults were enumerated following the methods described by Wari et al. [18]. In brief, twenty leaves per treatment were sampled every week. Eggs and nymphs (including 1st, 2nd, 3rd, and 4th instar nymphs) underneath the tomato leaves were counted using a Leica S6E stereo microscope mounted with a Leica KL300 LED illumination. *Bemisia tabaci* egg and nymph counts for the twenty leaves were pooled together and the average calculated to designate the *B. tabaci* egg and nymph counts per tomato leaf in a given treatment. The averages for each sampled week are treated as replicate for each treatment per annual study. One 200 cm^2^ yellow sticky trap (Arysta Lifesciences, Tokyo, Japan) was set in the middle of each treatment and collected after three days to determine *B. tabaci* adult population. Sticky trap setting and collection (together with leaves) were performed on weekly basis for 13 weeks. After leaf and sticky trap sampling, synthetic insecticides were applied in the positive control greenhouse treatment as per the application schedule in Appendix A (Table A1).

### 2.5. Statistical Analyses

*Nesidiocoris tenuis* and *B. tabaci* (eggs, nymphs and adults separately) count were tested for normality (Shapiro–Wilk test and Lilliefors test) and homogeneity (Bartlett’s Test) using an open-source software OpenStat (http://openstat.info/OpenStatMain.htm, accessed on 10 February 2021). The total number of *N. tenuis* (nymphs and adults) and the eggs, nymphs and adults of *B. tabaci* were transformed using ‘*x* + 1’ to meet the assumptions of analysis of variance (ANOVA). The effects of “treatment (hemp rope and banker plants)” and “sampling interval (weeks)” factors and their interaction on the population trends of *N. tenuis* on tomato were analyzed using univariate repeated-measures ANOVA. While the effect of *N. tenuis* on *B. tabaci* eggs, nymphs and adults was analyzed using a repeated-measures ANOVA model with treatment and sampling interval considered as fixed factors. *Nesidiocoris tenuis* distribution among the zones in the greenhouses between the hemp rope and banker plants treatments were analyzed using contingency chi-square test for two-way tables to test the relationship between treatments and zones. In addition, three-way contingency table for treatment, zones and weeks (sampling interval) was analyzed using log-linear regression model. Furthermore, standard deviations (SD) were calculated from averages of pooled data (40~48 tomato plants per greenhouse for *N. tenuis* counts per treatment, 10~12 tomato plants per zone for *N. tenuis* counts per zone, 20 tomato plant leaves per treatment for *B. tabaci* eggs and nymphs, and one 200 cm^2^ yellow sticky trap per treatment for *B. tabaci* adults) per sampled week from two annual studies to represent the difference between the years.

## 3. Results

### 3.1. Nesidiocoris tenuis Population Dynamics on Tomato Plants

**Figure 1:**
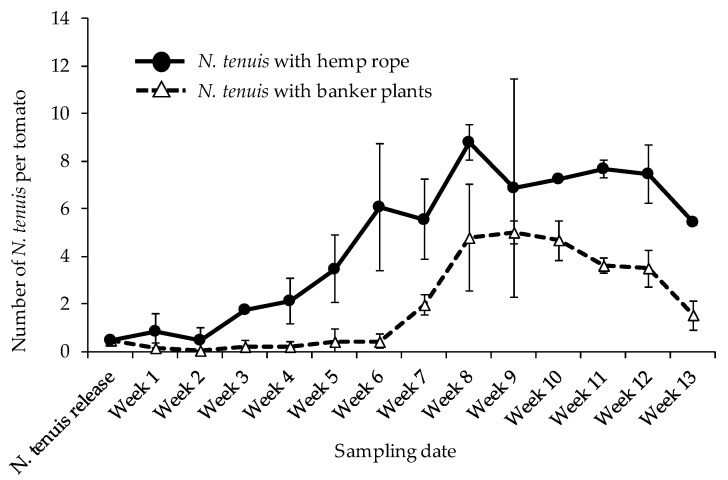
Mean (±SD) number of *Nesidiocoris tenuis* (inclusive of nymphs and adults) on tomato plants when augmented with hemp rope and banker plants.

A total of six weeks after *N. tenuis* was released onto the tomato plants with hemp rope, *N. tenuis* (inclusive of nymphs and adults) were observed to be gradually increasing in numbers per tomato plant. Significant increase could be seen on the fifth week from the start of the survey and subsequently peaking at week eight at 8.80 ± 0.76 individuals per tomato plant (Figure 1). Comparatively, *N. tenuis* trends on tomato plants in the banker plant treatment greenhouse was stationary until week 6 of the survey, then peaking gradually until reaching its highest peak at the ninth week with 5.03 ± 0.47 individuals (nymphs and adults inclusive) per tomato plants (Figure 1). Both hemp rope and banker plants promoted *N. tenuis* development on tomato plants. However, proliferation on tomato plants was delayed when *N. tenuis* was augmented with banker plants (Figure 1). A significant increase in *N. tenuis* during time post-release was observed on tomato plants in both treatments. However, the onset of the increase was significantly earlier in hemp rope treatment than in banker plants treatment, leading to the significant interaction in the sampling interval (*p* = 0.0001) (Table 1).

### 3.2. Impact of N. tenuis on B. tabaci Eggs, Nymphs, and Adults on Tomato Plants

**Figure 2:**
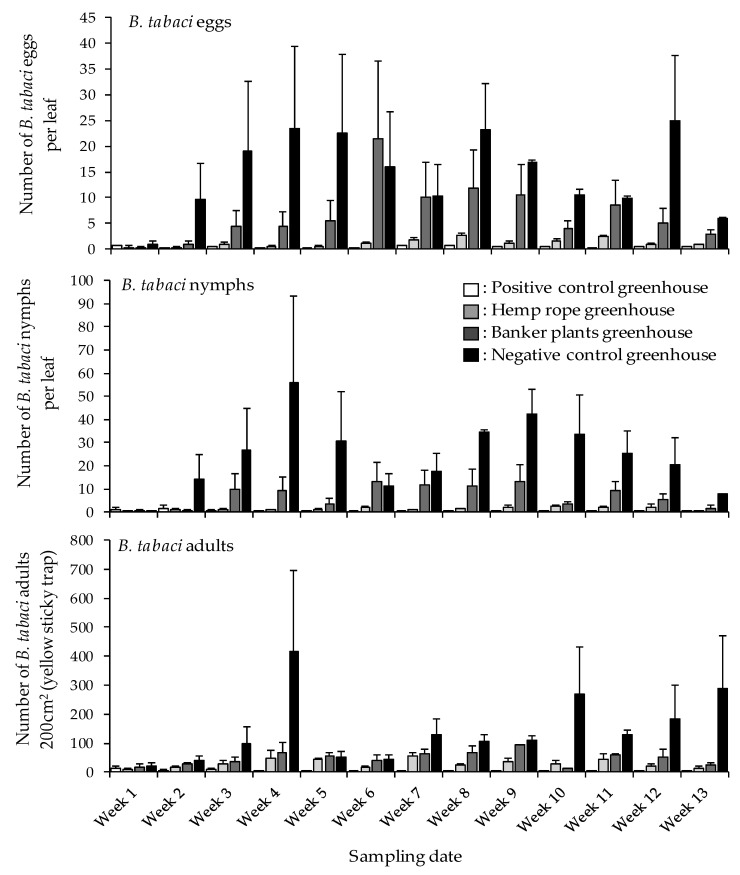
Mean (±SD) number of *Bemisia tabaci* eggs, nymphs, and adults in the four treatments; positive control greenhouse, hemp rope greenhouse, banker plant greenhouse, and negative control greenhouse.

To examine if *N. tenuis* augmented with hemp rope could impact the population of *B. tabaci*, population densities of *B. tabaci* eggs, nymphs, and adults were determined. Results showed that the interaction between treatments, sampling intervals (weeks) or between treatments and sampling intervals in the hemp rope, banker plants, positive and negative control treatments did not significantly impact the number of *B. tabaci* eggs on tomato plants under greenhouse conditions (Table 2). Similarly, the interaction between treatments, sampling intervals (weeks) or between treatments and sampling intervals in the hemp rope vs. banker and hemp rope vs. positive control did not significantly affect *B. tabaci* nymphs and adults. However, interaction between treatments (hemp rope vs. negative control) yielded significant differences in the population densities of *B. tabaci* nymphs and adult in hemp rope vs. negative control (*p* ≤ 0.001 and *p* = 0.003, respectively) (Table 2). Interaction between sampling intervals or between treatments and sampling intervals did not significantly affect *B. tabaci* nymphs and adults. These observations are reflective of the population densities of *B. tabaci* nymphs and adults between treatments shown in Figure 2 where *B. tabaci* nymphs and adults are shown to be considerably lower in the hemp rope, as well as positive control treatments compared to banker plants and negative control treatments. It can be deduced that the reduced number of *B. tabaci* nymphs in hemp rope treatment is the direct impact of *N. tenuis* while the reduced *B. tabaci* densities in positive control treatment is due to the aggressive insecticide application approach (Table A1).

### 3.3. Nesidiocoris tenuis Population Trends on Banker Plants

**Figure 3:**
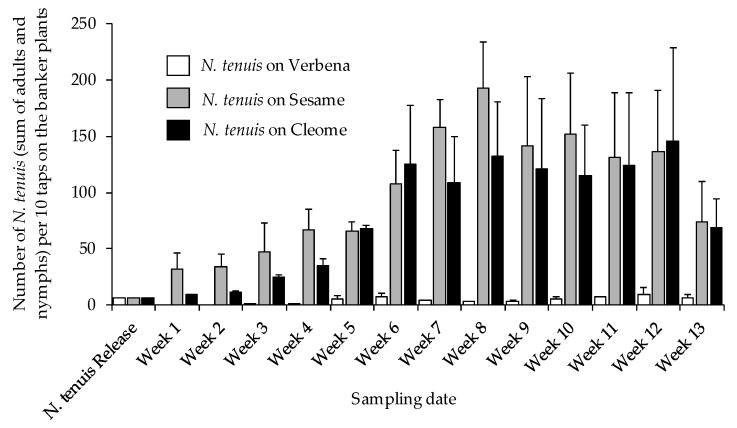
Mean (±SD) number of *Nesidiocoris tenuis* (including nymphs and adults) on banker plants; Verbena (*Verbena terena* cv. Tapian), Sesame (*Sesamum indicum*), and Cleome (*Cleome spinosa*).

Banker plants are known for their role in augmenting the growth and development of natural enemies by providing them with shelter, food, and a habitat to lay eggs and propagate. To avoid early damage by *N. tenuis* on tomato seedling, banker plants (verbena, sesame, and cleome) were used. To ensure that *N. tenuis* is present and is moving onto tomato plants after propagating on banker plants, population trends of *N. tenuis* on banker plants were determined. The results showed that the number of *N. tenuis* in the banker plants increased as the survey progressed. While cleome and sesame contributed larger quantities of *N. tenuis*, the same was not observed for the verbena (Figure 3).

### 3.4. Nesidiocoris tenuis Distribution among Tomato Plants

**Figure 4:**
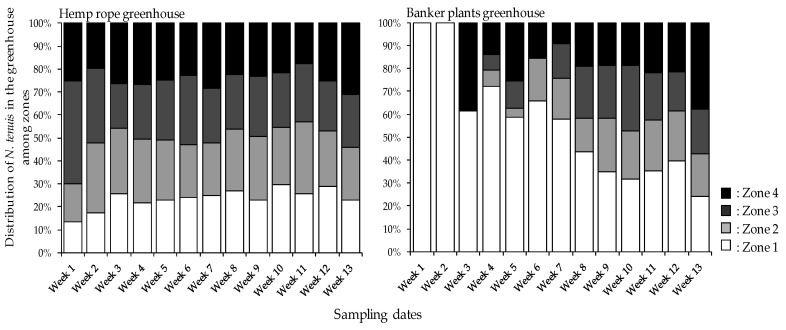
*Nesidiocoris tenuis* distribution on tomato plants in four zones when augmented with hemp rope and banker plants.

To determine if *N. tenuis* (both nymphs and adults) are scattered among the tomato plants in the greenhouse, we observed the distribution of the *N. tenuis* individuals on tomato plants. *Nesidiocoris tenuis* were observed to be equally distributed among the tomato plants two weeks from the start of the survey when augmented with hemp rope. However, *N. tenuis* in the banker plant treatment greenhouse were unevenly distributed among the zones (Figure 4). This is evident as *N. tenuis* counts were highly significantly different (*p* ≤ 0.001) between the hemp rope and banker plants treatment as well as between the zones from week one of the survey until week seven (Table 3). On the other hand, there was only a moderate correlation (*r* = 0.576, *r^2^* = 0.3295) between the treatments (hemp rope and banker plants), zones (zone 1, 2, 3 and 4) and weeks (sampling intervals from week 1 to 13) implying that while there were significant differences between the treatments and the zones in the start of the survey, the significant differences diminished as the survey continued. To determine if different growth stages of *N. tenuis* counts (adults and nymphs) would provide insights into such trends, *N. tenuis* adults and nymphs were analyzed separately. While significant differences (*p* ≤ 0.001) between the hemp rope and banker plants treatment as well as between the zones could be observed, significant difference in *N. tenuis* adults were observed until week 7 of the survey while nymphs a week later (Figure A1 and Table A1). This observation is of no surprise as the nymph emergence and or occurrences are expected to follow that of the adults. As expected, *N. tenuis* individuals clustered mainly in zone 1 in the first half of the study in the banker plant treatment as zone 1 was adjacent to the banker plants and progressively moved onto other zones as the study continued. On the other hand, *N. tenuis* were expected to scatter among the tomato plants in all zones in the hemp rope treatment. Presumably, the presence of the factitious diet during the release of *N. tenuis* assisted with their spread.

## 4. Discussion

*Nesidocoris tenuis* has been a success story in managing key pests of tomato crops in the Mediterranean basin as well as in Japan [6,19]. Releasing and maintaining the *N. tenuis* on tomato plants at a rate of 0.5 to 1 insect per plant over the whole cultivation period is usually recommended to minimize *N. tenuis*-mediated damages on tomato [8,9]. To minimize proliferation beyond the recommended rate, supplementing *N. tenuis* with banker plants such as sesame or verbena has been recommended and been practiced in the western part of Japan (9). The whole idea behind supplementing *N. tenuis* with banker plants is so that *N. tenuis* can proliferate on banker plants to the maximum number of individuals in a given time and can move to the tomato plants to prey on *B. tabaci*, *T. absoluta* and *T. palmi* when these prey are present. While relocation of *N. tenuis* from the banker plants to tomato plants is always guaranteed, distribution among all the tomato plants under the field conditions may be delayed and, thus, delay control of a given pest, as shown in our data (Figure 1 and Figure 4). A system that promotes natural enemy proliferation is therefore needed to enhance propagation, relocate, and spread among the cultivated crops.

Factitious prey is preferentially used in supplementing and augmenting biological control agents. Factitious prey can be used as a live specimen, frozen, irradiated, or lyophilized insects, mites, and crustaceans. *Artemia* cysts are live aquatic crustaceans having the potential to promote natural enemy growth and development [7,10,11,12,13,14,15,16,17]. Sugar has also been documented to nutritionally supplement *N. tenuis* [20]. Combining *Artemia* cysts with sugar, Owasi et al. [7] showed in vitro that life-history traits of *N. tenuis* could be improved and recommended for future studies. Building on the research by Owasi et al. [7], we showed under greenhouse conditions that *N. tenuis* proliferation was accelerated when *Artemia* cysts were supplemented with high fructose corn syrup and honey as sugar source. The increased proliferation thus encouraged the spread of *N. tenuis* among the tomato plants. As a result of the increase in proliferation and spread among tomato plants, the number of *B. tabaci* eggs and nymphs was suppressed as compared to the control treatment. With all variables constant, the use of *Artemia* cysts enhanced with high fructose corn syrup and honey as factitious dietary to augment *N. tenuis* seem promising for *B. tabaci* control in a protected system, as such in tomato production.

While successful *N. tenuis* proliferation, spread, and control of *B. tabaci* eggs and nymphs could be observed in the hemp rope supplemented greenhouse, an accelerated increase in *N. tenuis* individuals was also observed. According to Calvo et al. [8] and the *N. tenuis* “how to use” manual by NARO [9], 0.5 to 1 individual per two tomato plants is recommended. However, more than 0.5 to 1 *N. tenuis* insect per tomato plant (maximum of nine individuals per plant at the highest peak) was observed in our study. While it can be argued that the increased number of *N. tenuis* can be advantageous in managing pests, scarcity in prey numbers as a result of over-predation can result in plant-feeding. There are three models proposed by Gillespie and McGregor [21] to determine the feeding behavior of omnivorous Heteropterans; (i) the amount of plant-feeding decreases with increased prey feeding, (ii) the amount of plant-feeding increases with increased prey feeding, and (iii) the amount of plant-feeding is independent of the amount of prey feeding. Calvo et al. [8] observed that *N. tenuis*, a classic example of zoophytophagous insect that plant-feeds when animal feeds are scarce seemed to follow the first model. They further concluded that damage to tomato plants decreased in response to the greater availability of *B. tabaci*. Arno et al. [22] also made similar observations under laboratory conditions. However, in our study, necrotic rings were observed on the younger shoots of tomato plants (data not shown) even prey was still present. *Artemia* cysts are promising, in that they accelerate *N. tenuis* proliferation; however, a higher number of *N. tenuis* individuals on tomato plants may not only prey on the pests such as whiteflies but also supplementing themselves with nutrients through feeding on tomato plants, yielding plant damage.

*Artemia* cysts enhanced with high fructose corn syrup and honey are a cheap and cost-effective source of factitious supplementary dietary. Our study, under greenhouse conditions, further supports the in vitro studies by Owasi et al. [7] establishing *Artemia* cysts mixed with high fructose corn syrup and honey are equally efficient as banker plants in augmenting *N. tenuis* growth and development while improving their spread among the tomato plants. While an increase in *N. tenuis* numbers on tomato plants can improve pest control, an increase beyond recommended rates (0.5–1 individual per plant) can negatively impact the crop (tomato) growth and productivity. Further field studies are needed to optimize the use of *Artemia* cysts enhanced with high fructose corn syrup and honey in augmenting *N. tenuis* under both open and protected field settings, at the same time, management of *N. tenuis* using selective pesticides to minimize plant-feeding. These parameters, therefore, are required in the optimization and development of the Integrated Pest Management (IPM) system in tomato against whiteflies. Furthermore, this study was performed in two consecutive years in a 10 m by 5 m greenhouses. Multiple consecutive annual studies and in large scale systems is needed to categorically substantiate these results.

## 5. Conclusions

In the past decade, studies have been performed on the suitability of *Artemia* cysts as factitious supplementary dietary for natural enemies and/or predators under in vitro or semi-field conditions [7,10,11,13,14,15,16,17,23,24,25,26]. We showed under greenhouse conditions the *Artemia* cysts enhanced with high fructose corn syrup and honey could augment and improve the growth and development of *N. tenuis*. The improved growth and development can hypothetically enhance quicker settlement of the natural enemies on the crops thus, unsettling and decelerating the establishment of the intended pest before it can cause any further economic damages. The results in this study establish foundational data in building towards an IPM system integrating *N. tenuis*, *Artemia* cysts enhanced with isomerized sugar (high fructose corn syrup) and honey, and selective pesticides to control greenhouse pests.

## Figures and Tables

**Table 1:** Results of the univariate repeated-measures analysis on the population trends of *Nesidiocoris tenuis* on tomato plants as affected by treatments and the sampling interval, and the interaction of these two factors.

*N. tenuis* Trends on Tomato Plants	*F*	*d.f.*	*p*
Treatments	7.78	2, 49	0.0012
Sampling interval	4.65	12, 39	0.0001
Treatment x sampling interval	8.65	25, 26	0.0002
Week 1	1.11	24, 27	0.0516
Week 2	1.34	24, 27	0.0505
Week 3	9.16	24, 27	<0.0001
Week 4	9.13	24, 27	<0.0001
Week 5	8.39	24, 27	<0.0001
Week 6	6.47	24, 27	<0.0001
Week 7	6.34	24, 27	<0.0001
Week 8	1.50	24, 27	0.0513
Week 9	1.15	24, 27	0.0523
Week 10	4.79	24, 27	<0.0001
Week 11	5.71	24, 27	<0.0001
Week 12	6.65	24, 27	<0.0001
Week 13	9.22	24, 27	<0.0001

**Table 2:** Results of the repeated-measures ANOVA showing main effects and interactions of *Nesidiocoris tenuis* when augmented with hemp rope compared to banker plants, positive control (chemical control) and negative control (no means of pest control means) on densities of *Bemisia tabaci* eggs, nymphs and adults on tomato plants.

Experiment	Source	Response Variable								
		*B. tabaci* Eggs			*B. tabaci* Nymphs			*B. tabaci* Adults		
		*d.f.*	*F*	*p*	*d.f.*	*F*	*p*	*d.f.*	*F*	*p*
Hemp rope vs. Banker plants: Tomato + *N. tenuis* + *B. tabaci*	Treatment	1	0.95	0.508	1	1.12	0.482	1	2.04	0.389
	Sampling intervals	12	1.07	0.453	12	1.18	0.390	12	0.92	0.557
	Treatment x Sampling interval	12	0.94	0.541	12	1.28	0.339	12	0.93	0.546
Hemp rope vs. Positive Control: Tomato + *B. tabaci*	Treatment	1	13.93	0.167	1	3.26	0.322	1	36.59	0.104
	Sampling intervals	12	1.20	0.376	12	0.22	0.992	12	0.45	0.911
	Treatment x Sampling interval	12	1.84	0.153	12	0.97	0.524	12	0.88	0.585
Hemp rope vs. Negative Control: Tomato + *B. tabaci*	Treatment	1	3.99	0.298	1	31.96	**<0.001**	1	57,465.69	**0.003**
	Sampling intervals	12	0.42	0.928	12	0.41	0.537	12	0.47	0.899
	Treatment x Sampling interval	12	0.46	0.905	12	0.49	0.499	12	0.56	0.840

**Table 3:** Two-way chi-square tests for *Nesidiocoris tenuis* (sum of nymphs and adults) distribution between treatments (hemp rope and banker plant treatments) and zones (Zone 1, 2, 3 and 4) for each sampling intervals.

Treatment x Zones	χ2	*d.f.*	*p* Value
Week 1	142.61	1	<0.001
Week 2	131.58	1	<0.001
Week 3	58.67	1	<0.001
Week 4	52.64	1	<0.001
Week 5	37.09	1	<0.001
Week 6	48.79	1	<0.001
Week 7	25.56	1	<0.001
Week 8	7.39	1	0.061
Week 9	3.35	1	0.341
Week 10	1.11	1	0.776
Week 11	3.60	1	0.306
Week 12	2.54	1	0.468
Week 13	1.49	1	0.684

## Data Availability

Not applicable.

## Appendix A

**Table A1:** Two-way Chi-square tests for *Nesidiocoris tenuis* adults and nymph distribution between treatments (hemp rope and banker plant treatments) and zones (Zone 1, 2, 3 and 4) for each sampling intervals (weeks).

Treatment x Zones	*N. tenuis* Adults			*N. tenuis* Nymphs		
	χ2	*d.f.*	*p* Value	χ2	*d.f.*	*p* Value
Week 1	111.50	1	<0.001	162.50	1	<0.001
Week 2	117.28	1	<0.001	148.89	1	<0.001
Week 3	50.51	1	<0.001	60.85	1	<0.001
Week 4	38.05	1	<0.001	73.48	1	<0.001
Week 5	31.51	1	<0.001	60.34	1	<0.001
Week 6	43.41	1	<0.001	81.52	1	<0.001
Week 7	11.12	1	0.011	64.73	1	<0.001
Week 8	2.61	1	0.456	18.65	1	<0.001
Week 9	6.23	1	0.101	5.26	1	0.154
Week 10	1.91	1	0.592	2.19	1	0.534
Week 11	4.01	1	0.261	8.74	1	0.033
Week 12	1.11	1	0.775	5.97	1	0.113
Week 13	3.45	1	0.370	1.61	1	0.657

**Table A2:** Synthetic insecticide spraying schedule for the positive control treatment greenhouse for 2019 and 2020 studies.

2019				2020			
Application Week	Insecticide	IRAC Code	Application Rate *	Application Week	Insecticide	IRAC Code	Application Rate *
Week 1	Spinetoram	5	0.4 µL mL^−1^	Week 1	Spinetoram	5	0.4 µL mL^−1^
Week 2	Abamectin	6	1.0 µL mL^−1^	Week 2	Abamectin	6	1.0 µL mL^−1^
Week 3	Pyrifluquinazon	9B	0.25 mg mL^−1^	Week 3	Pyrifluquinazon	9B	0.25 mg mL^−1^
Week 4	Dinotefuran	4A	0.5 mg mL^−1^	Week 4	Dinotefuran	4A	0.5 mg mL^−1^
Week 5	Cyantraniliprole	28	0.5 µL mL^−1^	Week 5	Cyantraniliprole	28	0.5 µL mL^−1^
Week 6	Spinetoram	5	0.4 µL mL^−1^	Week 6	Spinetoram	5	0.4 µL mL^−1^
Week 7	Milbemectin	6	0.75 µL mL^−1^	Week 7	Pyrifluquinazon	9B	0.25 mg mL^−1^
Week 8	Pyrifluquinazon	9B	0.25 mg mL^−1^	Week 8	Milbemectin	6	0.75 µL mL^−1^
Week 9	Spinetoram	5	0.4 µL mL^−1^	Week 9	Cyantraniliprole	28	0.5 µL mL^−1^
Week 10	Dinotefuran	4A	0.5 mg mL^−1^	Week 10	Dinotefuran	4A	0.5 mg mL^−1^
Week 11	Lepimectin	6	0.5 µL mL^−1^	Week 11	Lepimectin	6	0.5 µL mL^−1^
Week 12	Flonicamid	29	0.5 mg mL^−1^	Week 12	Milbemectin	6	0.75 µL mL^−1^

*: Insecticides were mixed in a 15 L sprayer at the rates specified above.
